# Trends of Antihypertensive, Antidiabetic, and Nonsteroidal Anti-Inflammatory Drugs Use among the Health Workers Cohort Study, Mexico 2004 to 2018

**DOI:** 10.1155/2023/5555274

**Published:** 2023-11-21

**Authors:** Janinne Ortega-Montiel, Alejandra Montoya, René Soria-Saucedo, Katia Gallegos-Carrillo, Paula Ramírez-Palacios, Jorge Salmerón, Eduardo Salazar-Martínez

**Affiliations:** ^1^Population Health Research Centre, National Institute of Public Health, Cuernavaca, Mexico; ^2^Carlos Slim Foundation, Mexico City, Mexico; ^3^Higher University of San Andres, La Paz, Bolivia; ^4^Epidemiology and Health Services Research Unit, Mexican Institute of Social Security, Cuernavaca, Mexico; ^5^Research Centre in Policy, Population, and Health, School of Medicine, National Autonomous University of Mexico, Mexico City, Mexico

## Abstract

**Background:**

Hypertension and type 2 diabetes (T2D) are the most prevalent noncommunicable diseases in Mexico and worldwide. According to international practice management guidelines, the principal chronic management therapy is daily oral medication.

**Aim:**

We aim to describe the trends of antihypertensive, antidiabetic, and nonsteroidal anti-inflammatory (NSAID) drugs use among the Mexican adult population from 2004–2018.

**Methods:**

We analyzed data from the Health Workers Cohort Study (HWCS) for males and females aged >18 years. We calculated the prevalence of chronic diseases and utilization for every kind of antihypertensive, antidiabetic, and NSAIDs (measured by self-reported utilization) at baseline and two follow-ups (2004, 2010, and 2017). Trends were analyzed using Fisher's exact test.

**Results:**

Hypertension prevalence increased from 19.8 to 30.3%, higher than T2D prevalence from 7.0 to 12.8% through fourteen years of follow-up. Like the self-reported dual therapy, the proportion of patients using beta-blockers and angiotensin II receptor blockers increased. Regarding T2D, the prevalence of metformin utilization increased to 83.9%. The utilization of common NSAIDs, mainly for muscular pain, remained around 13 to 16%.

**Conclusions:**

Our findings showed a changing prevalence of drug utilization for hypertension and T2D between 2004 and 2018 and consistent use of NSAIDs in the adult Mexican population.

## 1. Introduction

Chronic diseases in adults such as hypertension [1], type 2 diabetes (T2D) [2], renal disease, and musculoskeletal conditions are the most common and high morbidity or comorbidity that represents a significant public health problem in adulthood's last decades, which determines the health status in the elderly, along with pharmacotherapy as the primary control and management treatment through different daily oral drug classes use [3].

Mexican current clinical practice guidelines agree with international guidelines for hypertension [4] and recommend initial pharmacologic treatment with angiotensin-converting enzyme inhibitors (ACE-I), angiotensin II receptor blockers (ARB II), calcium channel blockers (CCB), thiazide diuretics (TD), and beta-blockers (BB) are now recommended under specific indications as a combined therapy. On the other hand, clinical guidelines for pharmacologic treatment in type 2 diabetes [5] recommend metformin (biguanide) as first-line pharmacological treatment, followed by a second-line drug such as sulfonylureas, glucagon-like peptide (GLP-1) receptor agonists, sodium-glucose cotransporter-2 (SGLT2) inhibitors, and dipeptidyl peptidase 4 (DPP-4) inhibitors, among others. Pharmacologic treatment for both diseases can be prescribed as monotherapy (use of only one drug) or combined therapy (dual pill or two classes of drugs on individual use).

However, since the mid-2000s, blood pressure control and glycemic management treatment have considerably changed in medical institutions around the world [6–9], particularly in Mexico, due to the availability of drugs in medical institutions [10, 11]. For hypertension, ACE-I, ARB II, and BB are now the most common drugs used as initial drug treatment, leaving the use of TD as part of dual pills; also, for T2D, metformin took relevance and became the most antidiabetic drug used as a first-line drug, with the recent introduction to the institutional catalog of new kinds of antidiabetics as pioglitazone and sitagliptin.

Furthermore, half of the adult population reported having suffered a musculoskeletal condition in recent years [12], besides a high prevalence of self-medication [13] with nonsteroidal anti-inflammatory drugs (NSAIDs). In addition, the medical prescription for pain relief as an analgesic has increased in the last decades [14, 15], such as acetaminophen, ibuprofen, salicylic acid, and naproxen are the most common drugs used, with or without knowledge of side effects or interactions [16, 17].

Given these changes in the pharmacotherapy approaches, it is essential to examine the temporal trends to provide information about care patterns, drug disuse described as noneffective, frequent side effects caused or determinant of chronic health status in response to changes in drug utilization, and identify potential improvement areas, like the adoption of recent specific drug indications adequate to each patient on daily practice medicine for avoiding polypharmacy in adults.

### 1.1. Aim of the Study

We aimed to investigate prevalence trends in the use of antihypertensive and antidiabetic drugs in diagnosed participants and NSAIDs in all adult participants using data from the Health Workers Cohort Study.

## 2. Materials and Methods

### 2.1. Data Source

We used data from the Health Workers Cohort Study (HWCS) [18]. The HWCS is a prospective open cohort study composed of employees from the Mexican Institute of Social Security (IMSS) (acronym in Spanish) and their families in Cuernavaca. IMSS is one of Mexico's three leading public healthcare institutions, providing healthcare to around 70 million adults [19]. The cohort is occupationally diverse, including physicians, nurses and nurse assistants, social workers, management, administration, and cleaning personnel; these employees and their families had medical insurance and prescription drug coverage by the same institution. The Ethics Committee of the IMSS approved the study protocol; every participant provided informed consent. The HWCS had three data collection assessments: 2004–2006, 2010–2013, and 2017-2018.

### 2.2. Study Population

We analyzed data from the three assessments of the HWCS participants aged ≥18 years and older; from the baseline assessment, we included 9,522 adult participants; the second assessment of the cohort included 2,075 participants, and the third assessment included 1,299 participants.

### 2.3. Measurements

The participants completed an extensive self-administered questionnaire in the presence of a health expert for clarification, providing detailed information about their demographics, health status, and lifestyle. Available data included the birth date, education, marital and employment status, family medical history, prior chronic illness diagnoses, medication regular utilization, diet, physical activity, smoking and alcohol consumption, social support, and quality of life [20, 21]. Participants also reported information about the diagnosis year and several chronic conditions diagnosed by a physician, such as T2D, hypertension, dyslipidemia, cardiovascular diseases, and respiratory diseases, among many others.

The commonly prescribed medications used to treat hypertension and T2D regularly were the exposure to interest. The questionnaire applied to the entire sample (same questionnaire in the three assessments) included a single-item question aiming to evaluate the common drug utilization prevalence, where the question was: “Which drugs do you take? (Mark the drugs that you usually take at least twice a week)” and the response options included none, acetaminophen, NSAIDs, thiazide diuretics, beta-blockers, calcium channel blockers, and others antihypertensive drugs, acetylsalicylic acid, hypolipemic, hormonal, anxiolytics, antidiabetic (metformin), antidepressants drugs, among others. Antihypertensive drugs were classified into five major categories: ACE-I (e.g., captopril and enalapril), ARBs (e.g., losartan and candesartan), CCB (e.g., amlodipine and nifedipine), TD (e.g., chlortalidone and hydrochlorothiazide), and beta-blockers (e.g., metoprolol and propranolol), the example drugs in parenthesis were the most specific drug used for each category. We only had metformin, glibenclamide, and sitagliptin information regarding antidiabetic drugs.

After answering the questionnaire, participants were scheduled for an appointment within three months to take anthropometric and laboratory measurements. Body weight (kg) was measured with calibrated electronic scales (Tanita BC-533®) with a precision of 0.2 kg, with the participant wearing minimum clothing and no shoes. Height (mt) was measured using a conventional stadiometer (SECA 213®) with a precision of 1 mm, with the participants standing barefoot with their shoulders in a normal position. Body mass index (BMI, kg/m^2^) was calculated as weight in kilograms divided by the square of height in meters.

### 2.4. Statistical Analysis

We calculated the frequency and proportion of each characteristic of the study population (socio-demographic, job status, anthropometrics) and medical history, the prevalence of hypertension, T2D, and musculoskeletal disorders, among other comorbidities, for the three assessments, and percentages comparison to baseline assessment were conducted by Fisher's exact test.

For the conducted prevalence trends analysis, we included participants with self-reported hypertension diagnosed by a physician or antihypertensive drug use, those with self-reported T2D diagnosed by a physician or antidiabetic drugs, and those with self-reported NSAID use. Over the three assessments, we estimated the prevalence and 95% interval confidence of using antihypertensives, antidiabetics, and NSAID drugs. Comparison to baseline assessment was conducted by Fisher's exact test for each difference in antihypertensive, antidiabetic, and NSAID drug proportions [8]. A two-sided *P* value <0.05 was considered statistically significant. Stata 15 and StataCorp LP (College Station, TX, USA) statistical package were used for statistical analyses.

## 3. Results

Table 1 presents the cohort characteristics in 2004–2006, 2010–2013, and 2017-2018. The proportion of participants aged ≥60 years increased from 2004 through 2018 and reached 40%, as expected in an adult cohort study. Women proportion was higher than men in the three assessments (69.5% in 2004, 51.2% in 2010, and 75.2% in 2017, *p* < 0.0001). The prevalence of obesity increased from 19.4% to 26% from 2004 to 2017 (*p* < 0.0001). The education degree increased, and nurses' participation decreased across the study population. Between comorbidities, only renal chronic disease prevalence remained stable.

### 3.1. Trends in Antihypertensive Drugs

From 9,522, 2,975, and 1,299 participants of the HWCS in 2004, 2010, and 2017, respectively, the change in self-reported hypertension prevalence was from 1,882 (19.8%) to 725 (24.4%) and 394 (30.3%) (*p* < 0.0001), respectively; therefore, the patients with hypertension diagnosis were included in this analysis.

Of these patients, the proportion who referred no medical treatment during the following time decreased from 46.9% in 2006 to 23.6% in 2013, and 14% in 2018, *p* > 0.0001 (Table 1).

**Figure 1:**
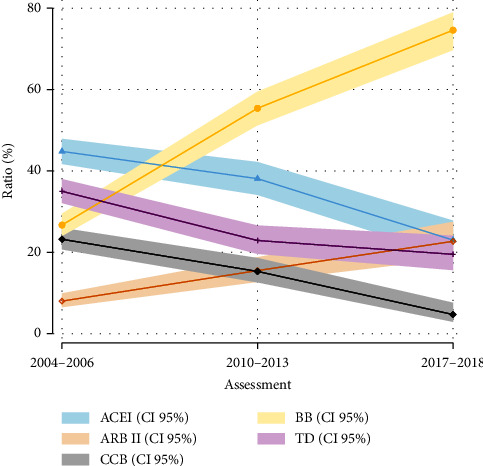
Trends in antihypertensive drug use in patients with hypertension, 2004–2006 to 2017-2018. ACE-I, angiotensin-converting enzyme inhibitor; ARB II, angiotensin II receptor blockers; CCB, calcium channel blockers; B-blocker, beta-blockers; TD; thiazide diuretics. Percentage use with 95% CI of antihypertensive drugs among participants with hypertension. We performed Fisher's exact test, and every percentage change was statistically significant (*p* < 0.01) with respect to the previous assessments, except for TD 2010–2013 to 2017-2018 (22.9% vs. 19.5%, *p* =0.12).

**Figure 2:**
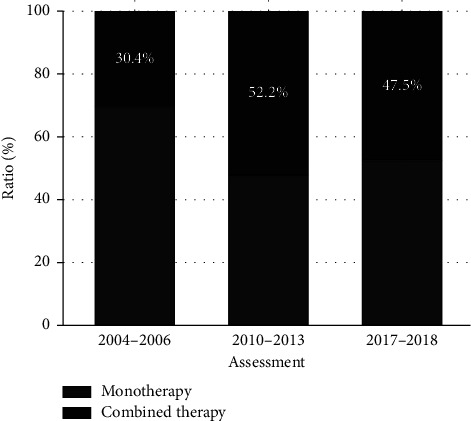
Classification of antihypertensive drugs used as monotherapy or dual therapy in the three assessments. Prevalence for the type of hypertension pharmacological therapy. Differences across the assessments (2010 and 2017 vs. 2004) were compared using Fisher's exact test. ^*∗*^Both changed percentages in 2010 were statistically significant (*p* < 0.01).

Beta-blockers and ARB II utilization increased during the fourteen years (26.6% to 74.6% *p* < 0.001 and 8% to 22.7% *p* < 0.001, respectively) (Table 2); meanwhile, the proportion of CCB utilization decreased from 23.2% to 4.7% *p* <  0.001, and ACE-I utilization from 44.7% to 23.0% *p* <  0.001. In contrast, the use of TD decreased in 2013 (34.9% to 23.9% *p* < 0.001) and then remained stable for the following years (Figure 1). Monotherapy was the most elected treatment, which decreased in 2013 (Figure 2).

### 3.2. Trends in Antidiabetic Drugs

The change in self-reported T2D prevalence was 668 (7.0%), 325 (10.9%), and 167 (12.8%) in participants of the HWCS in 2004, 2010, and 2017 (*p* < 0.0001), respectively. Monotherapy is the most prevalent treatment election within both diseases' hypertension and T2D (69.6%, 47.8%, and 52,5% in 2006, 2013, and 2018, respectively).

The use of any glucose-lowering medication increased by 27.3 (percentage points) from 2004 to 2018 (*p* < 0.001).

**Figure 3:**
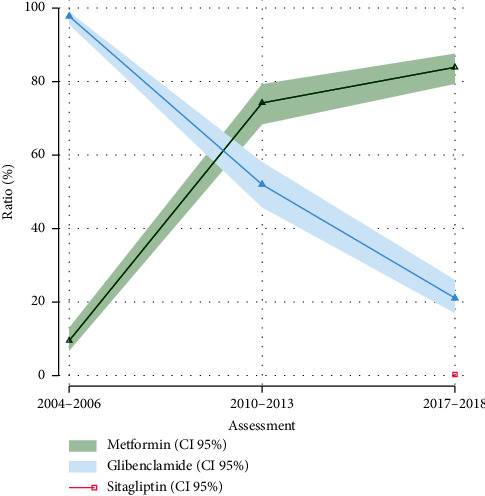
Trends in antidiabetic drug use in patients with T2D, 2004–2006 to 2017-2018. Percentage use with 95% CI of antidiabetic drugs among participants with T2D. Fisher's exact tests were performed; all percentage changes were statistically significant.

We observed a complete change in antidiabetic drug utilization trend during the study period characterized by a dramatic increase in the use of metformin (9.5% to 84%, *p* < 0.001, Table 2) and a marked decrease in the use of sulfonylureas (glibenclamide) (97.8% to 21.0%, *p* < 0.001). Until the third assessment data, the introduction of newer agents as sitagliptin (DPP-4 inhibitor) was evident in the institutional treatment (Figure 3).

### 3.3. Trends in Nonsteroidal Anti-Inflammatory Drugs

The overall percentage of people who reported musculoskeletal pain decreased from 2004 to 2010 (56.3% to 52.4%, *p* < 0.0001), then remained stable until 2017. The regular use of prescription analgesics increased from 2004 (45.6%) to 2017 (49.3%). Of the various analgesic classes, we showed a decrease in acetaminophen use, from 2.9% in 2006 to 2.1% in 2010 (*p* = 0.01).

**Figure 4:**
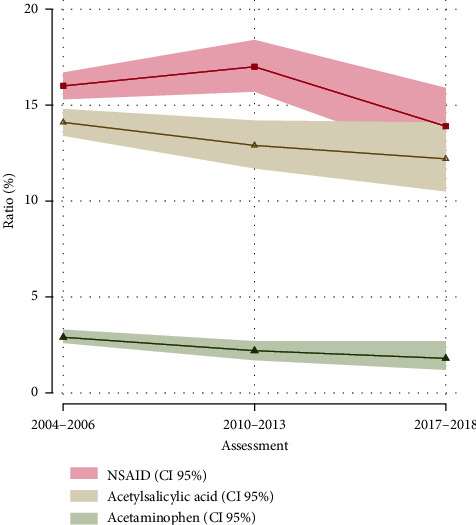
Trends in anti-inflammatories drugs use in overall cohort participants, 2004–2006 to 2017-2018. Percentage use with 95% CI of anti-inflammatories drugs in all participants. Fisher's exact tests were performed; three changes were statistically significant, anti-inflammatories (naproxen, ibuprofen, or diclofenac) from 2010–2013 to 2017-2018 (17.0% vs. 13.9%, *p* < 0.006), ASA from 2004–2006 to 2010–2013 (14.0% vs. 12.9%, *p* =0.05), and acetaminophen from 2004–2006 to 2010–2013 (2.9% vs. 2.1%, *p* < 0.01).

From 2004 to 2010, the use of one or more anti-inflammatory drugs (naproxen, ibuprofen, or diclofenac) slightly increased; then from 2010 to 2018, decreased from 17% to 14% in all participants (*p* =0.006) (Table 2). The decrease in acetylsalicylic acid (14% vs. 12.9%, *p* =0.05) and acetaminophen (2.9% vs. 2.1%, *p* =0.01) percentage use from 2004 to 2010 were statistically significant (Figure 4).

Among those regular NSAID users, the prevalence of reported self-medication practice decreased from 13.3% in 2006 to 8.24% in 2018.

## 4. Discussion

Our study showed changes in the prevalence trends in utilizing antihypertensive, antidiabetic, and NSAID through 14 years of study follow-up in HWCS adult participants. Independent of their health condition, we indeed observed an increase in common utilization of drugs in participants with a chronic disease.

In this study, the punctual prevalence of hypertension in the period study (2004–2006, 2010–2013, and 2017-2018) increased from 20% to 30%, following continuous results from the National Health and Nutrition Surveys of Mexico [10, 22]. The utilization of pharmaceutical treatment increased over 14 years among hypertensive participants. ACE-I, beta-blockers, and ARB II represent the most prevalent types of antihypertensive used in Mexican participants; these findings are congruent with previous studies and US [9], Japan [8, 23], Germany [24] findings, and market analysis [25]; although TD has been persistently used for the effectiveness on blood pressure control by reducing sodium and fluid retention; however, side effects are frequently presented at higher doses [26], even than they are essential as part of dual-pills polytherapy [27]; CCB plays a role in blood pressure too; however, it showed an important decreased in utilization from 23.2% to 4.7%.

The proportion of hypertensive patients treated with polytherapy was higher in 2013 than in 2006 and 2018 assessments (52.2%, 30.3%, and 47.5%, respectively), higher than reported by Chinese (24%) [28], lower than the US (56%–64.6%) [27, 29, 30], during approximately the same period.

Moreover, the burden of type 2 diabetes is still growing in Mexico, and the prevalence of T2D has constantly increased from 2000 to 2018, from 7.4% to 17%, with corresponding state-level variance [2, 31, 32]. Our results showed a similar positive trend (7% in 2004 to 12.8% in 2018). Although it is essential to see that among diabetic participants, the use of lower-glucose drugs increased by 27.4 percentage points, concerning the two most commonly used drugs (glibenclamide and metformin) here and around the world [32, 33], trends completely reversed through the years, the evidence has shown this change in other countries [6, 34, 35].

Nevertheless, we would like to discuss some crucial aspects of the health systems in Mexico. There are three major health systems: IMSS, which provides health care to 51% of the Mexican population; the Minister of Health; and the Institute of Security of Social Services for Employers (ISSSTE, Spanish acronymous). Public healthcare institutions purchase medications for their services and do not charge patients per product or event, but the availability and medication supply have been affected in the last few years [36–38]. Also, Mexico has one of the largest markets of pharmaceuticals in Latin America of patent medicines and generic drugs [39], and all kinds of antihypertensive, antidiabetic, and NSAID drugs are available for free, which means a prescription is not necessary; if any drug is not available at the institutional pharmacy, patients can quickly get it at any pharmaceutical dispensary [11].

In Mexico and other countries, the use and prescription of older classes of drugs such as sulfonylureas (glibenclamide specific) as monotherapy remained for decades [40–42], until we observed its evident decline. Suboptimal treatment should contribute to worsening diabetes control across generations. It was only in the half/final years of the 2000's decade that metformin took relevance in T2D treatment as a first-line treatment according to guidelines recommendation and until these days [43, 44].

Furthermore, in recent years, we observed that the use of newer second-line glucose-lowering medications, like DPP4-inhibitor (sitagliptin and linagliptin), which were included in 2015 to the national formulary coverage by the health institutional lists, among other classes like SGLT2 inhibitors and GLP-1 receptor analogs [45], their availability had increased in public health institutions but is still low overall for access and high cost. These similar trends have been shown in many countries like Colombia [46], US [7, 47], Korea [48], and Taiwan [49], among others. Even with the essential benefits of intensive T2D treatment to glucose control in these patients, monotherapy has been the most prevalent election treatment (92.7% in 2006, 73.4% in 2013, and 94% in 2018) in our study.

On the other hand, more than 50% of the cohort participants reported some musculoskeletal disorders and pain as the primary symptom, very similar to those shown by a Finnish cohort [12], according to the study time. Among 30% to 40% of the participants, reported taking common anti-inflammatory drugs (especially NSAIDs such as ibuprofen, naproxen, or diclofenac) prescribed by their physician or self-medicating for pain, similar to those reported by Ussai et al. [50]. These drugs as an anti-inflammatory or analgesic indication, are worldwide used by the adult population [14, 51–53], despite the common comorbidities existing in adult population [15, 54], the risk for polypharmacy [17], and drug-drug interactions [55].

The prescribed NSAIDs increased from 2004 to 2018 in our cohort (45.6% to 49.3%), higher than reported in the US [56]. However, we must take into account that, besides using NSAIDs for pain treatment, it has several different medical indications and easy access by free sale in Mexico, and acetaminophen is considered safe in most patients and remains constant in our time study.

For this study, 14 years of data were available, all collected rigorously and systematically by trained personnel who used standardized protocols. The list of drugs reported by the participants agreed with the national formulary coverage available at the IMSS.

Some limitations of this study need to be emphasized; the number of participants enrolled in each assessment decreased because of the focus on worksite available to following and monetary resources; these could lead to selection bias with overrepresentation of participants with some chronic disease diagnosis, limiting the external validity of the prevalence showed; also, we could not correlate drug self-report use with specific physician prescription of medical record history of participants; furthermore, we did not collect information on precise daily dose and medication adherence; drug utilization was self-reported, and patients' health literacy could impact their recognition of the drugs which can lead to underestimation of the drugs' utilization and misclassification. Access to these essential drugs depends on institutional pharmacy supply and entire filled prescriptions. However, these drugs are free to buy in Mexico, and many generic presentations are available.

## 5. Conclusions

The present study showed that the proportion of participants with hypertension using beta-blockers and ARB II increased. In contrast, TD and CCB utilization decreased during the follow-up time. As shown in other countries, the primary drug used as monotherapy for T2D has wholly changed. The use of NSAID in the study population remained constant across the three assessments without considering comorbidities, polytherapy, and age. In Mexico, given the fractional structure of the health system plus private medical attention, different studies made by some institutions like ours contribute to the knowledge of the pharmacoepidemiology of commonly used drugs for chronic diseases in the Mexican adult population; the integration of all this information could support changes in public policies for monitoring and regulation of drugs utilization.

## Figures and Tables

**Table 1:** Cohort sociodemographic and clinical characteristics across the three assessments.

Characteristic	2004–2006 *N* = 9,522	2010–2013 *N* = 2,975	*p* value	2017-2018 *N* = 1,299	*p* value
*Gender*					
Female	6,615 (69.5)	1524 (51.2)	0.0001	977 (75.2)	0.0001
Male	2,907 (30.5)	1451 (48.8)		322 (24.8)	

*Age categories*					
18–40 yr	4,324 (45.4)	888 (30.9)	0.0001	224 (17.3)	0.0001
41–60 yr	3,912 (41.1)	1,392 (48.5)		555 (42.9)	
61–80 yr	1,222 (12.8)	557 (19.4)		482 (37.2)	
>81 yr	64 (0.7)	35 (1.2)		34 (2.6)	

*Education degree*					
Elementary school or below	2194 (23.0)	610 (20.5)	0.0001	290 (22.3)	0.02
High school/technical trainee	1556 (16.3)	436 (14.6)		249 (19.1)	
Bachelor of above	3884 (40.8)	838 (28.2)		486 (37.4)	
Data not available	1888 (19.9)	1091 (36.7)		274 (21.2)	

*Job title*					
Physician	151 (1.6)	53 (1.8)	0.0001	25 (1.9)	0.0001
Nurse	465 (4.9)	76 (2.6)		34 (2.6)	
Administrative	930 (9.8)	125 (4.2)		53 (4.0)	

*BMI categories (kg/m* ^*2*^)					
Healthy weight (min—24.9)	3496 (38.8)	969 (35.9)	0.016	429 (33.6)	0.0001
Overweight (25.0–29.9)	3766 (41.8)	1160 (42.9)		517 (40.4)	
Obesity (30.0—max)	1756 (19.4)	572 (21.2)		332 (26)	

*Comorbidities*					
Hypertension	1882 (19.8)	725 (24.4)	0.0001	394 (30.3)	0.0001
No treatment	884 (47.0)	171 (23.6)	0.0001	55 (14.0)	0.0001
Medication treatment	998 (53.0)	554 (76.4)		339 (86.0)	
Type 2 diabetes	668 (7.0)	325 (10.9)	0.0001	167 (12.8)	0.0001
No treatment	309 (46.3)	73 (22.5)	0.0001	74 (19.0)	0.0001
Medication treatment	359 (53.7)	252 (77.5)		317 (81.0)	
Musculoskeletal disorders	5362 (56.3)	1559 (52.4)	0.0001	688 (52.9)	0.15
Medication pain treatment	1931 (36.0)	647 (41.5)	0.0001	327 (47.5)	0.0001
Chronic renal disease	75 (0.8)	16 (0.5)	0.098	7 (0.5)	0.21

BMI, body mass index. Variables showed as frequencies (Percentage). Comparison to baseline characteristics (2010 and 2017 vs 2004) were compared using Fisher's exact test.

**Table 2:** Percentage and 95% CI of utilization of each drug across the three assessments.

Drug	2004–2006		2010–2013		2017-2018	
	Percentage	CI 95%	Percentage	CI 95%	Percentage	CI 95%
*Antihypertensive*						
ACE-I	44.8%	41.7–47.9	38.1%	34.1–42.2	23.0%	18.8–27.8
ARB II	8.0%	6.5–9.9	15.5%	12.7–18.8	22.7%	18.5–27.5
CCB	23.2%	20.7–26.0	15.3%	12.6–18.6	4.7%	2.9–7.6
B-blocker	26.7%	23.9–29.5	55.4%	51.2–59.5	74.6%	69.7–79.0
TD	35.0%	32.0–38.0	22.9%	19.6–26.6	19.5%	15.6–24.1

*Antidiabetic*						
Metformin	9.5%	6.8–13.0	74.2%	68.4–79.3	83.9%	79.4–87.6
Glibenclamide	97.8%	95.6–98.9	52.0%	45.8–58.1	21.1%	16.9–26.0
Sitagliptin					0.9%	0.3–2.9

*NSAIDs*						
Anti-inflammatories	16.0%	15.3–16.7	17.0%	15.7–18.4	13.9%	12.1–15.9
Acetylsalicylic acid	14.1%	13.4–14.8	12.9%	11.8–14.2	12.2%	10.5–14.1
Acetaminophen	2.9%	2.6–3.3	2.2%	1.7–2.7	1.8%	1.2–2.7

ACE-I, angiotensin-converting enzyme inhibitor; ARB II, angiotensin II receptor blockers; CCB, calcium channel blockers; B-blocker, beta-blockers; TD, thiazide diuretics; NSAID, nonsteroidal anti-inflammatory drug. Estimates of proportions, standard errors, and 95% CI were made by the values in each drug category.

## Data Availability

The data that support the findings of this study are available from the corresponding author upon reasonable request.

## Abbreviations

T2D: Type 2 diabetes
ACE-I: Angiotensin-converting enzyme inhibitors
ARB II: Angiotensin II receptor blockers
CCB: Calcium channel blockers
BB: Beta-blockers
TD: Thiazide diuretics
NSAID: Nonsteroidal anti-inflammatory drugs
HWCS: Health Workers Cohort Study
IMSS: Mexican Institute of Social Security
ISSSTE: Institute of Security of Social Services for Employers
GLP-1: Glucagon-like peptide 1 receptor agonist
SGLT2: Sodium-glucose cotransporter 2 inhibitors
BMI: Body mass index
DPP-4: Dipeptidyl peptidase 4 inhibitors.
